# Cross-scale neutral ecology and the maintenance of biodiversity

**DOI:** 10.1038/s41598-018-27712-7

**Published:** 2018-07-05

**Authors:** James P. O’Dwyer, Stephen J. Cornell

**Affiliations:** 1 Department of Plant Biology, University of Illinois, Urbana, IL USA; 2 Institute of Integrative Biology, University of Liverpool, Liverpool, L69 7ZB UK

## Abstract

One of the first successes of neutral ecology was to predict realistically-broad distributions of rare and abundant species. However, it has remained an outstanding theoretical challenge to describe how this distribution of abundances changes with spatial scale, and this gap has hampered attempts to use observed species abundances as a way to quantify what non-neutral processes are needed to fully explain observed patterns. To address this, we introduce a new formulation of spatial neutral biodiversity theory and derive analytical predictions for the way abundance distributions change with scale. For tropical forest data where neutrality has been extensively tested before now, we apply this approach and identify an incompatibility between neutral fits at regional and local scales. We use this approach derive a sharp quantification of what remains to be explained by non-neutral processes at the local scale, setting a quantitative target for more general models for the maintenance of biodiversity.

## Introduction

Neutral biodiversity theory has become one of the most tested paradigms of macroecology^1–4^. It combines the ecological mechanisms of birth, death, competition, speciation, and spatial dispersal to make predictions for ecological patterns, and makes manifest the belief that the many differences between species may not be critical for successfully predicting large-scale, aggregated phenomena. Subsequent studies have expanded on the original neutral approach^5,6^, generalizing the theory to include life history^7,8^, fitness differences^9–11^ and multiple modes of speciation^12,13^. But at the core of this theory there is a missing link: we lack a complete picture of how neutral predictions change with spatial scale.

Censusing of species abundances at the scale of hectares has provided a tractable approach to test neutral predictions. At these local scales we would expect dispersal limitation to significantly affect the distribution of species abundances alongside birth, death, and competition, leading to departures from the log series distribution of abundances that neutral theory predicts at the largest scales^1,14,15^. But existing neutral predictions for local community abundances are based on spatially-implicit models, meaning that the process of dispersal is not modeled explicitly, and is instead treated by fitting a parameter in a sampling process. Because there are differences between this spatially-implicit approximation and the full, spatially-explicit neutral processes^16^, it has been unclear whether neutrality is consistent with observed local community species abundances or not. Spatially explicit neutral approaches have been developed by modelling dispersal with a dispersal kernel, including numerical simulations^1,17–20^; hybrid approaches where non-spatial parameters are fitted to a spatially-explicit simulations^16,21^; the limit of very short-scale dispersal^22^; a focus on predicting pairwise correlations in species composition^23–25^; phenomenological models^26^; and analytical approaches that make statistical assumptions which are violated in real communities^27,28^. In short, each has some drawbacks, approximations, or limitations in applicability, so that while spatially explicit models make more realistic assumptions, only the spatially implicit theory has so far been compared exhaustively to empirical abundance patterns.

In this paper, we address this gap by introducing a new mathematical formulation of the spatial theory of neutral biodiversity, derived using the backward equation formulation of stochastic processes. While an exact solution of these equations is not available due to non-linearities, we have identified an accurate approximation scheme which we test extensively using spatially-explicit numerical simulations. These new results allow us to generate a set of predictions that connect both local observations and regional data, in a way that is faithful to neutral model assumptions across this range of scales. We subsequently parametrize the neutral model using sparse, regional and continental-scale observations, and go on to test whether it is then consistent with distributions of abundance at the local scale. We focus on data that has already been fitted using spatially-implicit models, to see whether our spatially-explicit approach deviates from these earlier results.

Combining our modeling approach with data from these multiple scales, we find that neutrality alone significantly underestimates local species diversity, and also deviates from the observed distribution of rare and abundant species. Our intuition might have been that dispersal and neutrality would lead to many rare, transient species, which disperse into a local community and quickly drop out before proliferating. In fact, our spatial neutral prediction dramatically under-predicts the observed number of rare species. This indicates that local stabilizing mechanisms are likely important to understand and accurately predict local patterns of biodiversity^29–31^, and precisely quantifies what remains for these approaches to explain.

## Model

Our model is based around the neutral assumptions of intrinsic birth and mortality rates that are identical across all species, in addition to symmetric competition for a single resource, which we approximate using the mean field approach^10^. This is also known as a non-zero sum formulation^32^ because the total community size is allowed to fluctuate around an average value. We assume that individuals are sessile and dispersal takes place at birth, though as we show in the Supplementary Information the same species-area curve and species-abundance distribution would occur if the organism moved during its lifetime. The resulting model is an assemblage of ecologically identical species, with a constant, total density across space and time when in steady state. New species enter the community via speciation, which occurs at a fixed per capita rate, and hence a fixed rate per unit time and area. All species eventually leave the community due to extinction. So the model reduces to a set of independent populations, beginning their existence with a single individual, and proliferating transiently across space. Meanwhile, we would like to predict the probability that a focal species has a given number of individuals in our sample location in the present day.

In our Supplementary Information we derive the following backward equation (so-called because we look ‘backwards’ from the present day, as explained in our Supplementary materials) to characterize these dynamics and this observable:

1 $$\begin{array}{rcl}\frac{{\partial }P(k,A,x,y,t)}{\partial t} & = & (b-\nu )\sum _{m=0}^{k}P(k-m,A,x,y,t)P(m,A,x,y,t)\\  &  & +\,b{\sigma }^{2}\sum _{m=0}^{k}P(k-m,A,x,y,t)\\  &  & \times \{\frac{{\partial }^{2}}{\partial {x}^{2}}P(m,A,x,y,t)+\frac{{\partial }^{2}}{\partial {y}^{2}}P(m,A,x,y,t)\}\\  &  & -\mathrm{(2}b-\nu )P(k,A,x,y,t)+b{\delta }_{k\mathrm{,0}}.\end{array}$$

In this equation, *P*(*k*, *A*, *x*, *y*, *t*) is the probability that a single individual at time 0, at a spatial location defined by coordinates *x* and *y*, will have *k* conspecific descendants in a sample region of area *A*, after a time *t* has elapsed. We can derive patterns of species abundance from this quantity because, if the individual at time 0 is the founding individual of the species, then *P*(*k*, *A*, *x*, *y*, *t*) is the total number of members of this species in the sampling region at time *t*. This equation describes how *P* changes in time and space due to the processes of birth (with associated dispersal) and death, and is derived by considering how the lineage starting from a single individual can change in a short time interval. The first term represents birth of a conspecific (which takes place at rate (*b* − *ν*)), and is bilinear in *P* because birth leads to two independent lineages which can contribute descendants to the sampling region. The sum over *m* represents the different combinations of lineages that lead to a total *k* descendants in the focal region. Offspring of a different species are produced at rate *ν*, but these do not contribute to *P*(*k*, *A*, *x*, *y*, *t*). The second term contains a Laplacian to represent the fact that offspring are distributed at a random distance, proportional to the parameter *σ*, from their parent. The term *bδ*_*k*,0_ (using the Kronecker *δ* symbol, which is equal to one if *k* = 0, and zero otherwise) represents the fact that a death event (occuring at rate *b*) causes a lineage containing a single individual to go extinct (in which case the only possibility is *k* = 0).The linear term, whose prefactor (2*b* − *ν*) is the sum of the birth and death rates, represents the fact that either a birth or a death event will change the number of descendants.

From the definition of the quantity *P*(*k*, *A*, *x*, *y*, 0), the appropriate initial condition for this equation is simply that at *t* = 0, if the location (*x*, *y*) is inside the sample area *A*, then *P*(*k*, *A*, *x*, *y*, 0) = *δ*_*k*,1_, again using the Kronecker *δ* symbol. Conversely, if (*x*, *y*) is outside the sample area, *P*(*k*, *A*, *x*, *y*, 0) = *δ*_*k*,0_. We have chosen the sample area to be circular, and centered at the point 0,0 in two-dimensional space. Assuming for the time being that we can solve Eq. (1) for *P*(*k*, *A*, *x*, *y*, *t*), then we immediately have a community level prediction for the average number of species with exactly abundance *k* in a sample area *A*:

2 $$S(k,A)=\nu \rho {\int }_{-\infty }^{\infty }dx{\int }_{-\infty }^{\infty }dy{\int }_{0}^{\infty }dt\,P(k,A,x,y,t),$$

where *ρ* is the constant average total density across space. However, solving Eq. (1) with the appropriate initial condition is non-trivial, due to the quadratic terms in *P*, which derive from the birth process, and we do not know of any closed-form solution. This non-linearity is the essence of why this is is a difficult problem, and is also reflected in the challenge of finding exact solutions in the corresponding forward-in-time, field theory version of this model^27^.

## Model Solutions

### Species-area curve

In our Supplementary Information we introduce an approximation scheme to linearize Eq. (1), with different linearizations applying in different regions of the landscape. As a special case of Eq. (2), we first focus on solutions for the Species-area curve, which counts the total number of distinct species (with any value of *k* > 0) as *A* increases, in this case for a circular sample region. We find the following approximate solution for this relationship:

3 $$\begin{array}{rcl}S(A) & = & \rho {\nu }_{eff}A\\  &  & +\,\frac{2\rho \sqrt{A\pi {\sigma }^{2}}(1-{\nu }_{{\rm{eff}}}){I}_{1}(\frac{\sqrt{A}}{\sqrt{\pi {\sigma }^{2}}})}{\frac{1}{\sqrt{{\nu }_{{\rm{eff}}}}}{I}_{1}(\frac{\sqrt{A}}{\sqrt{\pi {\sigma }^{2}}})\frac{{K}_{0}(\sqrt{A{\nu }_{{\rm{eff}}}/\pi {\sigma }^{2}})}{{K}_{1}(\sqrt{A{\nu }_{{\rm{eff}}}/\pi {\sigma }^{2}})}+{I}_{0}(\frac{\sqrt{A}}{\sqrt{\pi {\sigma }^{2}}})}\end{array}$$

**Figure 1:**
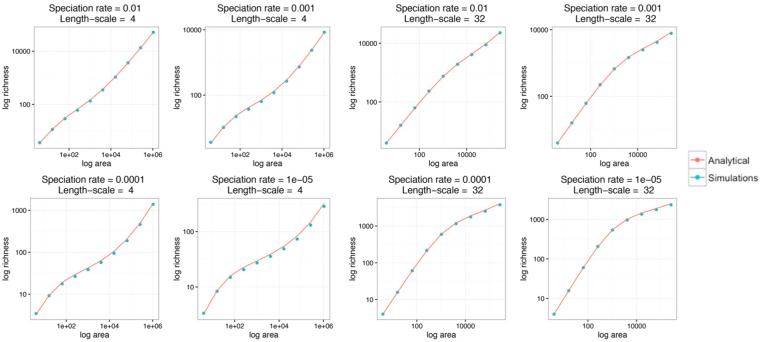
The species-area curve. We show a comparison between species richness as a function of sampled area for our analytical approximation to spatial neutral theory, compared with numerical simulations^17^. Over this range of values of speciation rate $(\frac{{\nu }}{{b}})$ and dispersal length-scale (*σ* in the main text), we see quantitative agreement between our approximation method and these earlier numerical results.

In this solution, we have used the short-hand ${\nu }_{{\rm{eff}}}=\frac{\nu }{b-\nu }\,\mathrm{log}(b/\nu )$, but no new parameters have been introduced, while *I*_*n*_ and *K*_*n*_ are modified Bessel functions. Note that only the per capita, per generation speciation rate, *ν*/*b* enters this solution, and so the rates *b* and *ν* do not independently affect the Species-area curve. How well does this approximation work? In Fig. 1 we demonstrate the agreement between theoretical and simulated curves over a range of speciation rates and values of *σ*.

At small areas, with $A\ll \pi {\sigma }^{2}$, both simulations and theoretical results give $S(A)\simeq \rho A$, i.e. where most new individuals belong to distinct species as the sample area is increased. At large areas, $A\gg \pi {\sigma }^{2}/{\nu }_{{\rm{eff}}}$, both simulations and theoretical results approach $S(A)\simeq \rho {\nu }_{{\rm{eff}}}A$, so that richness again increases linearly with area, but with a smaller overall coefficient. In between these extremes, we also see good agreement between the simulated and theoretical curves. The transition between large and intermediate scales has been modeled before, by making various phenomenological assumptions about species range shapes and distributions^33,34^. Here we can see that explicitly the first correction to large-scale linear behavior is proportional to $\sqrt{A}$, identical to these earlier results^33^, so that at intermediate to large scales:

4 $$S(A)\simeq \rho {\nu }_{{\rm{eff}}}A+\frac{2\rho \sqrt{A{\nu }_{{\rm{eff}}}\pi {\sigma }^{2}}}{\sqrt{{\nu }_{{\rm{eff}}}}+1},$$

again only valid when $A{\nu }_{{\rm{eff}}}/\pi {\sigma }^{2}\gg 1$. This agreement is non-trivial, given that the shape of any given neutral species range will not satisfy the simplifying assumptions (of circularity or convexity) made in the phenomenological approaches. Finally, the intermediate region as a whole has been fitted to empirical data drawn from across many taxa and enviroments using a power law^35^, and our resuls show that in neutral theory the power law SAC can only ever be an approximate description.

### Spatial Scaling of the Species Abundance Distribution

**Figure 2:**
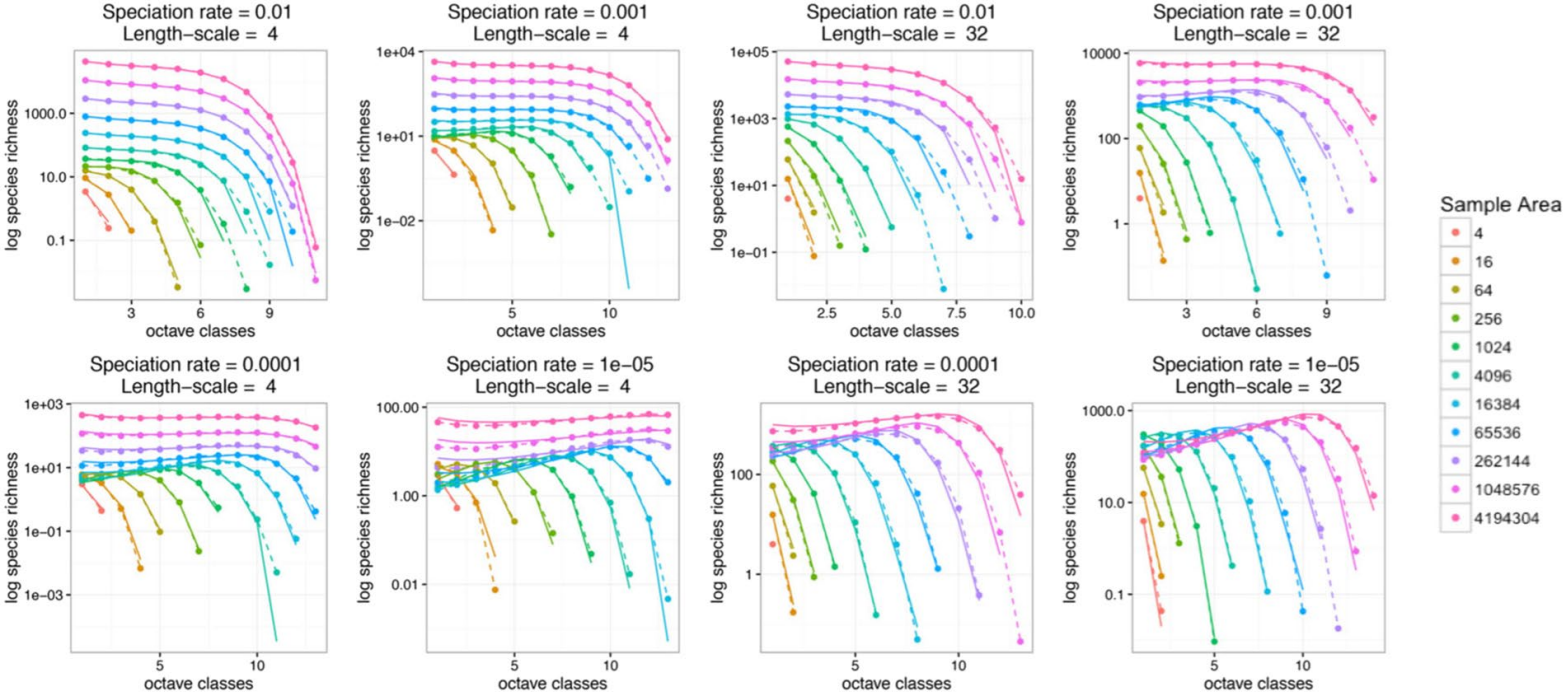
The species-abundance distribution. We test our approximation over a range of speciation rates, *ν*/*b* (and two different dispersal length-scale, *σ*), by comparing the predictions using Eq. (5) with numerical simulations^19^. Our results show good (though not perfect, due to both our approximations and the details of the numerical simulation) agreement over this range of parameter values.

We now apply the same approximation method to solve for the species abundance distribution, *S*(*k*, *A*), given by Eq. (2). Our solution is expressed in terms of the generating function, ${\rm{\Psi }}(z,A)={\sum }_{k\,=\,1}^{\infty }S(k,A){z}^{k}$, and we supply R code to extract the SAD itself from this generating function, following the method of^36^. Our solution is given by:

5 $$\begin{array}{rcl}{\rm{\Psi }}(z,A) & = & S(A)-\rho f(z)A\\  &  & +\,\frac{\frac{\mathrm{2(}f(z)-\mathrm{(1}-z))}{\sqrt{h(z)}}\rho \sqrt{A\pi {\sigma }^{2}}\,{I}_{1}(\sqrt{\frac{h(z)A}{\pi {\sigma }^{2}}})}{{I}_{0}(\sqrt{\frac{Ah(z)}{\pi {\sigma }^{2}}})+\sqrt{\frac{1-z}{f(z)}}\frac{{K}_{0}(\sqrt{\frac{Af(z)h(z)}{\mathrm{(1}-z)\pi {\sigma }^{2}}})}{{K}_{1}(\sqrt{\frac{Af(z)h(z)}{\mathrm{(1}-z)\pi {\sigma }^{2}}})}{I}_{1}(\sqrt{\frac{Ah(z)}{\pi {\sigma }^{2}}})}\end{array}$$

where we have defined the functions $f(z)=\frac{\nu }{b-\nu }\,\mathrm{log}\,[\frac{b-(b-\nu )z}{\nu }]$ and *h*(*z*) = 1 − *z*(1 − *ν*/*b*) for ease of notation, and *S*(*A*) is given by Eq. (3). While finding the Species-area curve is already a promising step, matching the full species abundance distribution as a function of area is a much sterner test for our approximation scheme. In Fig. 2, we show that our solution closely matches earlier numerical simulations over a range of speciation rates *ν*, values of dispersal length-scale, *σ*, and sample areas.

This expression for Ψ displays the properties of species abundance distributions that have previously been found by simulations of spatial neutral models^19^. First, when *A* is very large, the third term becomes much smaller than the second term, so the generating function is approximately *ρf*(*z*)*A*. Expanding in powers of *z*, we find in this limit

6 $$S(k,A)\propto \frac{{(1-\frac{\nu }{b})}^{n}}{n},$$

which is a Fisher logseries with diversity parameter $\alpha =1-\frac{\nu }{b}$.

Second, the species-abundance distributions display the “universality” noted by Rosindell and Cornell^19^. While the expression for Ψ depends on all four quantities *z*, *A*, *ν*/*b*, and *σ*, in Appendix 2.3 we show in that, when the speciation rate is small (*ν*/*b* → 0), it reduces to an expression that depends only on the two combinations *Z* = (1 − *z*)*b*/*ν* and *Y* = *Aν*/(*bσ*^2^). We also show in Appendix 2.3 that this is not limited to our approximation, but is also a property of the exact solution to the backward equation. We further show in Appendix 2.3 that this is equivalent to the species abundance distribution taking the scaling form $S(k,A)=\nu \tilde{S}(k\nu ,A\nu /{\sigma }^{2})$. This confirms analytically that species abundance distributions for spatial neutral models form a single-parameter family of curves, which extends the universality described by Storch *et al*.^34^ for species-area curves and endemics-area curves.

## Application to Tropical Forest Communities

Now armed with a spatially-explicit prediction for the species abundance distribution, we test whether the observed distribution of tree species abundances at the Barro Colorado Island 50 ha plot (BCI) is consistent with a neutral model where parameters are fixed independently of the plot-scale counts. Due to its high diversity and regular and comprehensive census, this plot has often been a testing ground for theoretical explanations of biodiversity patterns. It has also been extensively compared to the spatially-implicit neutral predictions, which have closely matched the observed abundance distribution^37–39^, although even early on it was emphasized that it may be difficult to distinguish neutral fits from alternatives with the same number of parameters^40,41^. Taking our alternative route, how should we determine the parameters of our spatially-explicit model? Density *ρ* is straightforward to estimate, and we could conceivably match the dispersal length-scale *σ* using inverse modeling and seed-trap data^42^. However, the speciation rate *ν* would be extremely challenging to measure directly, even to the extent that it is well-defined^12,13^.

Here we take a different approach, leveraging the methods and results of earlier studies focusing on large-scale spatial correlation functions^23,24^. These papers focus on the two-point spatial correlation function, known as *F*(*r*), the probability that two trees sampled at a separation *r* from each other are conspecifics. For spatial neutral theory, it has already been shown^23,27^ that this function takes the following form at large spatial separations:

7 $$F(r)=\frac{1}{\rho \pi {\sigma }^{2}}{K}_{0}(\frac{r\sqrt{2\nu /b}}{\sigma })\mathrm{.}$$

Using this result, and data from trees with diameter >10 cm in 34 1 ha plots in Panama (separated by values of *r* between ~0.5 and ~50 km), Condit *et al*.^24^ obtained parameter fits of *σ* = 40.2 *m* and *ν*/*b* = 5.10^*−*8^. These fitted values used the observed density of $\rho \simeq 0.04\,{m}^{-2}$. While speciation would be difficult to estimate independently of this fit, this value of *σ* is similar to those obtained from seed-trap data^42^.

**Figure 3:**
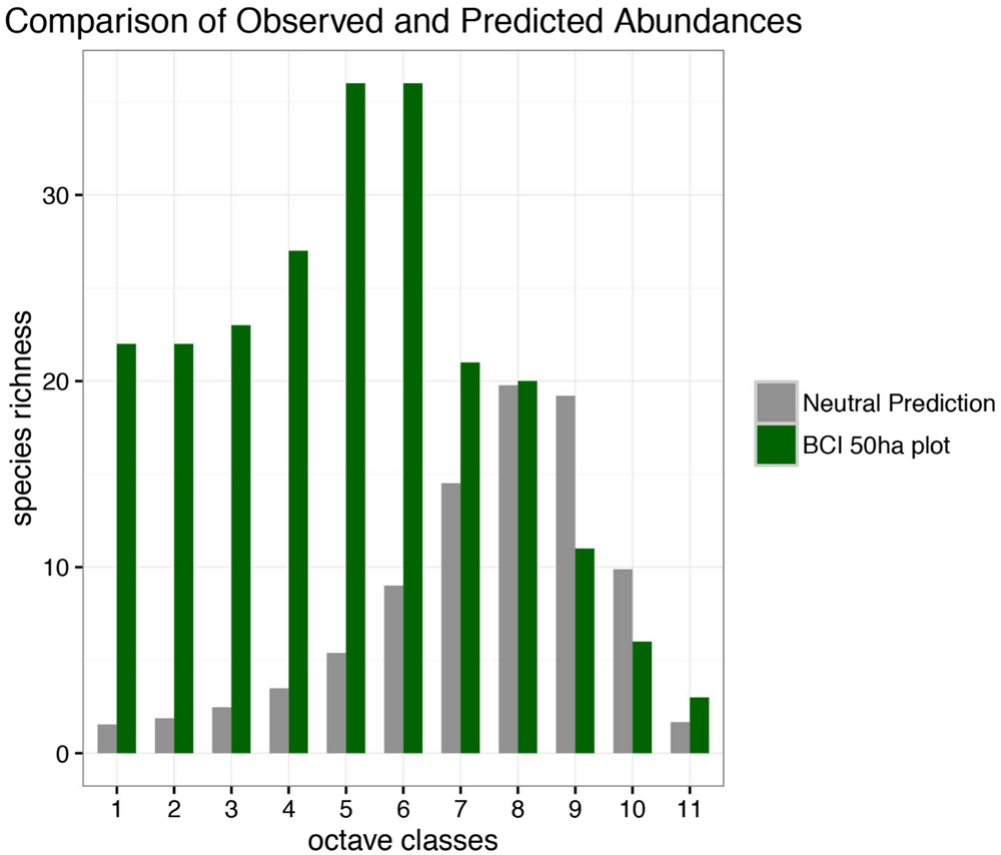
Neutral predictions at BCI. This comparison demonstrates the discrepancies between neutral predictions and the observed data at the 50 ha plot on Barro Colorado Island^43–45^. Neutral predictions are generated by fitting our spatial neutral model using large-scale data reported and analyzed in^24^. The results show that these large-scale fits produce a local-scale prediction for species abundances that both underestimates local species richness, compared with observed data, and also skews abundances from rare to more abundant species.

With these parameters fixed, we can test whether these large-scale data are consistent with the observed distribution of species abundance at the 50 ha plot scale^43–45^. Figure 3 demonstrates that the spatial neutral model severely underestimates diversity at the 50 ha scale, by approximately a factor of two. It also skews the distribution of species abundances towards more dominant species, with singleton species (those with just one stem >10 cm in the plot) underestimated by a factor of around twenty compared with observed counts. We also acknowledge the huge variation in fitted speciation rates across different regions using large-scale plot data–for example, South American plots fitted in^24^ led to much lower speciation rates than the data from Panama. To allow for the possibility that the large-scale Panama data is skewed in some way, in Fig. S1 we use the parameters fitted using Ecuadorian forest data, but find the same general pattern: an underestimate of overal local species richness at the 50 ha scale, alongside a skew away from rare species. We emphasize that while we are only looking at one plot, but this is a data set where spatially-implicit neutral predictions had already passed a series of tests, and so it is important to see whether these hold up when the neutral model is spatially-explicit. In summary, the formulation of spatial neutral theory we have considered here allows us to show that local abundances are not consistent with the parameters inferred from large-scale data.

## Discussion

Neutral theory has most often been formulated in a spatially-implicit way, so that local species abundance distributions depend on two free parameters characterizing the influx from a larger (but unmeasured) regional community^1,37^. These parameters, known as the fundamental diversity parameter, *θ*, and dispersal limitation parameter, *m*, respectively determine the richness of this larger community and then the rate of immigration from the regional to the local scale. It is certainly difficult to estimate the richness of this larger community, and hence *θ*, and while some model approaches have attempted to connect the *m* to explicit mechanisms of dispersal^21,46^, this matching only works in certain idealized limits. It has therefore been difficult to know the values of these two fitted parameters *θ* and *m* are biologically reasonable or not, even when the neutral theory successfully matches the distribution of species abundances in a local community.

We have introduced a new formulation of spatially-explicit, stochastic biodiversity theory that complements and extends the predictions of earlier approaches^17,23,27^. Making predictions from our model reduces to the solution of a non-linear partial differential equation, and while it is unlikely that this equation has a closed form solution, we identified an approximation scheme which closely matches the quantitative results of numerical simulations reported in previous work. The underlying model is essentially the same as considered in^27^, but the new formulation leads to a much better approximation of macroecological patterns (see Supplementary Information for a further discussion of the unrealistic approximations used in the ‘forward-in-time’ approach of^27^). We focused here on predictions for the species-area curve, and for the distribution of species abundances as a function of spatial scale. The latter prediction is a key advance over earlier formulations of neutral theory, as it allows us to test whether neutral theory matches observed abundance distributions without tuning parameters to fit this data.

It is uncontroversial to say that neutrality is an incomplete description of any given natural system. Instead, neutrality provides a starting point from which we might hope to infer the importance of non-neutral processes. The species abundance distribution has been largely written off as an approach to achieving this, in part because spatially-implicit neutral models are flexible enough to fit a vast range of different local abundance distributions. In applying our spatially-explicit methodology to Panamanian tropical forest data, we in part rehabilitate the species abundance distribution as a diagnostic for what is missing from the neutral explanation, in an approach consistent with previous calls to test multiple patterns simultaneously, rather than just species abundances alone^47^. Specifically, by fitting neutral parameters using large-scale large-scale pairwise correlation data, we identified a mismatch between those fitted parameters and local community abundances, under the neutral assumption: by fitting neutral parameters using large-scale data for the pairwise-similarity of widely separated plots, we were able to show that the corresponding neutral prediction for species abundances underestimates diversity at the 50 ha scale, and dramatically skews the distribution of abundances away from rare species. Conversely, we could in principle tune the neutral parameters to more closely match the 50 ha scale data, but at the expense of explaining large-scale spatial correlations poorly. Overall, this shows that neutral demographic processes, combined with Gaussian dispersal limitation alone, are unlikely to explain the maintenance of diversity at the both plot scale and regional scale.

Our goal in this study was not to identify what specific mechanisms could be added to the neutral dynamics to explain the maintainence of observed distributions of species abundances. However, there are several likely ways to resolve this mismatch, and our analysis now opens up the possibility of quantifying what kinds of additional ecological mechanisms provide the best explanation. Very generally, the skew towards rare species in the empirical data can be explained by the presence of stabilizing mechanisms at the local scale. Stabilization can arise from density-dependent interactions, perhaps in turn driven by plant-soil feedbacks^29,30^, which act to reduce both local dominance and extirpation. An alternative is that neutral models can still explain the presence of these rare species, but that we need to consider so-called “fat-tailed” dispersal, where the probability of dispersing a given distance from a parent tree drops off relatively slowly with distance^18,23,48^. Our results raise a challenge to either of these explanations for rare diversity in tropical forests. For example, if plant-soil feedbacks explain this combination of patterns, can we quantify exactly how strong and at what spatial scales these mechanisms must act? Similarly, can we quantify exactly what type of long-distance dispersal, if any, can explain the same patterns? Building on the development of this spatial model to include more general processes will provide a sharp, quantitative test of whether a given proposed mechanism is consistent with observations.

Neutrality has perhaps been tested more than any other single theory of biodiversity. This scrutiny has ranged across decadal fluctuations^49–51^ and evolutionary timescales^52–56^, and across taxonomic groups and environments^37,55,57–59^. In this manuscript, we show that in terms of the patterns where it has seen greatest success, species abundance distributions, we are seeing discrepancies between the theoretical predictions and observed data. On the other hand, the precise formulation of the neutral theory is exactly what makes it possible to perform these quantitative tests. While the presence of species differences and local niche structure has also been extensively tested, it has rarely been possible to translate the existence of these mechanisms into quantitative predictions for biogeographical patterns, like the distribution of species abundances as a function of spatial scale. The approach we have taken and discrepancies we have identified may therefore serve to motivate new, and more accurate, models of biodiversity, taking us a step closer to identifying precisely what mechanisms do and do not matter for the prediction of biodiversity patterns^60,61^.

## Electronic supplementary material

Supplementary Information: Methods and Derivations
