# Supplementary material for: Cross-scale neutral ecology and the maintenance of biodiversity
Source: Sci Rep. 2018 Jul 5;8:10200. doi: 10.1038/s41598-018-27712-7 (PMC6033888; doi:10.1038/s41598-018-27712-7)
Supplement: Supplementary file 1 — Supplementary Information: Methods and Derivations [file 41598_2018_27712_MOESM1_ESM.pdf]

Cross-scale neutral ecology and the maintenance of biodiversity: Methods and Derivations

James P. O'Dwyer [1], Stephen J. Cornell [2],
1 Department of Plant Biology, University of Illinois, Urbana IL USA
2 Institute of Integrative Biology, University of Liverpool, Liverpool L69 7ZB, UK

1 Backward Equation Derivation

We consider sessile individuals in d-dimensional space that give birth to conspecifics at a rate $(b-\nu)$ and die at rate b. Offspring are distributed relative to their parents according to a dispersal kernel B, i.e. the probability density that an offspring of an individual at position r is located at a position r' is $B(r-r')$. We assume that there are no density dependent interactions, so that there is no zero-sum rule and that species (and, more generally, lineages) do not interact. The difference between the birth and death rates represents the production of offspring that speciated (to a novel species) at birth.

We define $P(k,A,r,t)$ as the probability that a single individual at position r at time t_0 has exactly k conspecific descendants in a region A at time $t+t_0$ (the dynamics are translationally invariant in time, so this probability does not depend on t_0). We consider a single individual at time time 0, and enumerate the possibilities for its lineage during a small ensuing interval Δt, and the consequent value of $P(k,A,r,t)$:

Process	Probability	Probability of k descendants in A at time t
Nothing	$1-(2b-\nu)\Delta t$	$P(k,A,r,t-\Delta t)$
Death	$b\Delta t$	$\delta_{k,0}$
Birth, offspring at r'	$(b-\nu)\Delta t\, B(r-r')dr'$	$\sum_{m=0}^{k} P(m,A,r,t-\Delta t)P(k-m,A,r,t-\Delta t)$

If the individual dies, then there will be zero descendants ($\delta_{ij}[=1$ if $i=j$, 0 otherwise] is the Kroneker delta). If the individual gives birth, then there are two statistically independent lineages, and the total number of descendants in A is the sum of the number of descendants from the two lineages.

Combining these three three mutually exclusive possibilities gives

$$P(k,A,r,t) = (1-(2b-\nu)\Delta t)\, P(k,A,r,t-\Delta t) + b\Delta t \delta_{k,0}$$
$$+ (b-\nu)\Delta t \sum_{m=0}^{k} \int P(m,A,r',t-\Delta t) P(k-m,A,r,t-\Delta t) B(r-r') dr'. \quad (1)$$

Taking the limit of Δt going to zero gives:

$$\frac{\partial P(k,A,r,t)}{\partial t} = -(2b-\nu) P(k,A,r,t) + b\delta_{k,0}$$
$$+ (b-\nu) \sum_{m=0}^{k} \int P(m,A,r',t) P(k-m,A,r,t) B(r-r') dr', \quad (2)$$

where the integral runs over all d-dimensional space.

We assume that the dispersal kernel B decays to zero rapidly enough that $P(k-m,A,r,t)$ varies much more slowly in space, and perform a Taylor expansion for $P(m,A,r',t)$ so that

$$\int P(m,A,r',t) B(r-r') dr' = \int B(r'-r) \left\{ 1 + (r'-r)\cdot\nabla + \frac{((r'-r)\cdot\nabla)^2}{2} + \dots \right\} P(m,A,r,t) dr'$$
$$\approx P(m,A,r,t) + K\nabla^2 P(m,A,r,t),$$

where $K = \frac{1}{2}\int (r'-r)^2 B(r'-r) dr'$ and we have assumed that B is normalised so that $\int B(r'-r) dr' = 1$, and has rotational symmetry so that $\int (r'-r) B(r'-r) dr' = 0$. The backward equation can then be written as

$$\frac{\partial P(k,A,r,t)}{\partial t} = -(2b-\nu) P(k,A,r,t) + b\delta_{k,0}$$
$$+ (b-\nu) \sum_{m=0}^{k} P(k-m,A,r,t) \left\{ P(m,A,r,t) + K\nabla^2 P(m,A,r,t) \right\} \quad (3)$$

This equation is to be solved for $t > 0$, with initial condition $P(k,A,r,t) = \delta_{k,1}$ if r is inside the region A, and zero otherwise, representing the fact that if $t = 0$ the lineage consists of the founding individual only.

Finally, we define the generating function of P as:

$$G(z,r,t,A) = \sum_{k=1}^{\infty} z^k P(k,A,r,t) \quad (4)$$

so that

$$\frac{\partial G}{\partial t} = (-2b+\nu)\, G + (b-\nu) G^2 + b + DG\nabla^2 G, \quad (5)$$

where $D = K(b - \nu)$. If $\nu \ll b$, then $D = b\sigma^2$, where σ^2 is twice the variance of the dispersal kernel. The boundary condition is that $G(z, r, 0, A)$ is $= z$ if r is within the sampling region defined by A (e.g. a line segment of length L in the one dimensional problem, and an area of a particular geometry in the 2-dimensional case).

A similar backward equation can be derived for species that, rather than being sessile and dispersing at birth, performed random walks. This equation turns out to be identical to eqn. (5) except that the factor G in front of the Laplacian is replaced by 1. As discussed later, this factor does not affect the Species Area Curve or Species Abundance Distribution in the biologically relevant limit $\nu \ll b$.

2 Solutions

2.1 Species Area Curve

We now consider an approximation method to find solutions of Eq. (5). Considering spatial dimension 1, first we define:

$$\phi(x, t, L) = 1 - G(x, 0, t, L) \tag{6}$$

so that ϕ satisfies:

$$\frac{\partial \phi}{\partial t} = -\nu\phi - (b - \nu)\phi^2 + D(1 - \phi)\frac{\partial^2 \phi}{\partial x^2}. \tag{7}$$

with an initial condition $\phi(x, t = 0, L) = R(x, L)$, where $R(x, L)$ is a rectangular function, equal to zero for $x < -L/2$ and $x > L/2$, and equal to one for $-L/2 < x < L/2$.

The function $\phi(x, t)$ is the probability that an individual appearing in a speciation event at a time t in the past, and at location x, will have one or more descendants in the focal region between $-L/2$ and $+L/2$ in the present day. In order to derive the Species-Area relationship from this probability distribution (where 'area' indicates the one-dimensional length of the focal region, L), we need to integrate over all speciation events, which in the neutral model occur at a rate $\nu\rho$ per unit time per unit area, where ρ is the equilibrium density of individuals in space. Hence, our goal is to derive a solution for:

$$S(L) = \nu\rho \int_{-\infty}^{\infty} dx \int_{0}^{\infty} dt \; \phi(x, t, L). \tag{8}$$

Due to the nonlinearity in Eq. (7), solving for $\phi(x, t, L)$ exactly does not seem tractable. But the initial and final conditions for ϕ suggest a linear approximation, if we treat $\phi^2(x, t, L) \simeq \phi(x, t, L)R(x, L)$. While true at $t = 0$, and true at late times when $\phi \to 0$, this does not hold for general t, and with this approximation for $S(L)$ we would underestimate the number of species at large values of L.

The problem is clear—at intermediate times, $\phi(x,t,L)$ will be non-zero outside of the focal region, and will interpolate between one and zero in side the focal region.

We therefore handle this discrepancy by approximating $\phi^2(x,t,L)$ as $\phi^2(x,t,L) = \phi(x,t,L)$ while x is within the focal region, and outside of the focal region we set $\phi^2(x,t,L) \propto \phi(x,t,L)$ with a (we expect small) constant of proportionality to be determined. In addition, we replace the factor $(1-\phi)$ in front of the Laplacian by 1, because when $\nu \ll b$ the dominant contribution to the integral that determined S comes from large values of t, by which time $\phi \ll 1$. This leads to an equation of the form:

$$\frac{\partial \phi}{\partial t} = -b\nu_{\text{eff}}\phi - b(1-\nu_{\text{eff}})\phi R(x,L) + D\frac{\partial^2 \phi}{\partial x^2}. \tag{9}$$

So in fact, we have two equations to solve, as the approximation we have used leads to different equations inside and outside of the focal region defined by L:

$$\begin{aligned} \frac{\partial \phi_{\text{in}}}{\partial t} &= -b\phi_{\text{in}} + D\frac{\partial^2 \phi_{\text{in}}}{\partial x^2} \\ \frac{\partial \phi_{\text{out}}}{\partial t} &= -b\nu_{\text{eff}}\phi_{\text{out}} + D\frac{\partial^2 \phi_{\text{out}}}{\partial x^2} \end{aligned} \tag{10}$$

We can now integrate over time, before solving these equations as a function of space, to obtain:

$$\begin{aligned} -b\nu_{\text{eff}}\rho &= -b\Phi_{\text{in}} + D\frac{\partial^2 \Phi_{\text{in}}}{\partial x^2} \\ 0 &= -b\nu_{\text{eff}}\Phi_{\text{out}} + D\frac{\partial^2 \Phi_{\text{out}}}{\partial x^2} \end{aligned} \tag{11}$$

where $\Phi_{\text{in}}(x,L) = \nu_{\text{eff}}\rho \int_0^\infty dt\ \phi_{\text{in}}(x,t,L)$ and $\Phi_{\text{out}}(x,L) = \nu_{\text{eff}}\rho \int_0^\infty dt\ \phi_{\text{out}}(x,t,L)$, respectively. Note that we have also introduced a new, effective rate of introduction of new species per unit space and time, $b\nu_{\text{eff}}\rho$, instead of $\nu\rho$, for consistency at small values of L with the term $\nu_{\text{eff}}\phi_{\text{out}}$ in Eq. (11). It may seem like we have introduced a free parameter or parameters by allowing for ν_{eff}, but in fact this effective rate is fixed by the large scale behavior, i.e. as $L \to \infty$. In this limit,

$$-\nu_{\text{eff}}\rho = -\Phi_{\text{in}} \tag{12}$$

which leads to a solution

$$S(L \to \infty) = \rho L \nu_{\text{eff}}. \tag{13}$$

So in order to match the standard neutral result for a well-mixed community, at large scales we have that

$$\nu_{\text{eff}}(\nu) = -\frac{\nu}{b-\nu}\log(\nu/b) \tag{14}$$

with no free parameters.

We can solve the pair of equations (11) by imposing that there is no singular behavior, that Φ_{out} asymptotes to zero for large x, and that at the boundaries $\pm L/2$ both Φ_{out} and Φ_{in} and their first

derivatives match. The result is:

$$\Phi_{\text{in}}(x,L) = \nu_{\text{eff}}\rho\left(1 - \frac{\cosh(mx)}{\frac{1}{\sqrt{\nu_{\text{eff}}}}\sinh(mL/2)+\cosh(mL/2)}\right)$$
$$\Phi_{\text{out}}(x,L) = \rho\frac{e^{-m|x-L/2|}\sinh(mL/2)}{\frac{1}{\sqrt{\nu_{\text{eff}}}}\sinh(mL/2)+\cosh(mL/2)} \tag{15}$$

where we have defined the inverse length-scale $m = \sqrt{b/D} = 1/\sigma$ which is related to the standard deviation of the dispersal distance. We now integrate over space to obtain our approximate prediction for the one-dimensional Species-Area relationship.

$$S = \nu_{\text{eff}}\rho\left[L + \frac{2(b/\nu_{\text{eff}}-1)}{m}\frac{\tanh(mL/2)}{\frac{1}{\sqrt{\nu_{\text{eff}}}}\tanh(mL/2)+1}\right] \tag{16}$$

In the following subsections we will show that despite our approximation, this provides an extremely accurate prediction of the relationship across a broad range of areas.

We now provide the corresponding result in two spatial dimensions. We apply exactly the same approximation, but where now we interpret $R(x,y,L)$ as the 'top-hat' function, which is equal to one inside a circular region of radius L, and is equal to zero outside. We then solve for functions inside and outside of this circular region:

$$-b\nu_{\text{eff}}\rho = -b\Phi_{\text{in}} + D\nabla^2\Phi_{\text{in}}$$
$$0 = -b\nu_{\text{eff}}\Phi_{\text{out}} + D\nabla^2\Phi_{\text{out}} \tag{17}$$

This leads to solutions which depend only on a radial coordinate, r (distance from the origin), and not on the corresponding polar coordinate:

$$\Phi_{\text{in}}(r,L) = \nu_{\text{eff}}\rho\left(1 - \frac{I_0(mr)}{\frac{1}{\sqrt{\nu_{\text{eff}}}}I_1(mL)\frac{K_0\left(mL\sqrt{\nu_{\text{eff}}}\right)}{K_1\left(mL\sqrt{\nu_{\text{eff}}}\right)}+I_0(mL)}\right)$$
$$\Phi_{\text{out}}(r,L) = \rho\frac{\sqrt{\nu_{\text{eff}}}\,I_1(mL)\frac{K_0\left(mr\sqrt{\nu_{\text{eff}}}\right)}{K_1\left(mL\sqrt{\nu_{\text{eff}}}\right)}}{\frac{1}{\sqrt{\nu_{\text{eff}}}}I_1(mL)\frac{K_0\left(mL\sqrt{\nu_{\text{eff}}}\right)}{K_1\left(mL\sqrt{\nu_{\text{eff}}}\right)}+I_0(mL)} \tag{18}$$

and integrating over all space we find:

$$S(\text{radius}=L) = \nu_{\text{eff}}\rho\left[\pi L^2 + \frac{2\pi L(1/\nu_{\text{eff}}-1)}{m}\frac{I_1(mL)}{\frac{1}{\sqrt{\nu_{\text{eff}}}}I_1(mL)\frac{K_0(m\sqrt{\nu_{\text{eff}}}L)}{K_1(m\sqrt{\nu_{\text{eff}}}L)}+I_0(mL)}\right] \tag{19}$$

where again $\nu_{\text{eff}} = -\frac{\nu}{1-\nu}\log(\nu/b)$. In terms of sample area $A = \pi L^2$ we can rewrite this as:

$$S(A) = \nu_{\text{eff}}\rho\left[A + \frac{2\sqrt{\pi}\sqrt{A}(1/\nu_{\text{eff}}-1)}{m}\frac{I_1(\frac{m\sqrt{A}}{\sqrt{\pi}})}{\frac{1}{\sqrt{\nu_{\text{eff}}}}I_1(\frac{m\sqrt{A}}{\sqrt{\pi}})\frac{K_0(m\sqrt{A\nu_{\text{eff}}/\pi})}{K_1(m\sqrt{A\nu_{\text{eff}}/\pi})}+I_0(\frac{m\sqrt{A}}{\sqrt{\pi}})}\right] \tag{20}$$

In the main text we replaced the inverse length-scale m with a length-scale $\sigma = 1/m$, but both can be related directly to the parameter D we introduced in the formulation of this problem.

2.2 Species Abundance Distribution

We now consider the same kind of approximation method to find solutions of Eq. (5), but instead of considering just total species richness, we define (again first considering the one-dimensional case):

$$\phi(x, z, t, L) = 1 - G(x, z, t, L) \tag{21}$$

so that ϕ again satisfies:

$$\frac{\partial \phi}{\partial t} = -\nu\phi - (b - \nu)\phi^2 + D(1 - \phi)\frac{\partial^2 \phi}{\partial x^2}. \tag{22}$$

with an initial condition $\phi(x, z, t = 0, L) = (1 - z)R(x, L)$, where $R(x, L)$ is the same rectangular function as above. To obtain the Species Abundance Distribution, we need to integrate over all speciation events, which in the neutral model occur at a rate $\nu\rho$ per unit time per unit area, where ρ is the equilibrium density of individuals in space. Hence, our goal is to derive a solution for:

$$\Psi(z, L) = S(L) - \nu\rho \int_{-\infty}^{\infty} dx \int_{0}^{\infty} dt \; \phi(x, t, L). \tag{23}$$

where using this definition, $\Psi(z, L)$ is related to the Species Abundance Distribution in a sample taken from the region of size L by

$$\Psi(z, L) = \sum_{k=1}^{\infty} S(k, L) z^k. \tag{24}$$

We now extend our previous approximation for the species area curve. We follow the same principle to set $\phi^2(x, z, t, L) \simeq (1 - z)\phi(x, z, t, L)$ when x is within the focal region, while outside of the focal region, we set $\phi^2(x, z, t, L) = g(z)\phi(x, z, t, L)$ for a function $g(z)$ to be determined by the requirement that we match the known behavior at large values of L. We again replace the factor $(1 - \phi)$ in front of the Laplacian by 1, as this makes a vanishing difference when $\nu \to 0$. This leads to an equation of the form:

$$\frac{\partial \phi}{\partial t} = -g(z)\phi - (b(1 - z) + \nu z - g(z))\phi R(x, L) + D\frac{\partial^2 \phi}{\partial x^2}. \tag{25}$$

Again, we have two equations to solve, as this approximation leads to different equations inside and outside of the focal region defined by L:

$$\begin{aligned} \frac{\partial \phi_{\text{in}}}{\partial t} &= -(b(1 - z) + \nu z)\phi_{\text{in}} + D\frac{\partial^2 \phi_{\text{in}}}{\partial x^2} \\ \frac{\partial \phi_{\text{out}}}{\partial t} &= -bg(z)\phi_{\text{out}} + D\frac{\partial^2 \phi_{\text{out}}}{\partial x^2} \end{aligned} \tag{26}$$

We can now integrate over time, before solving these equations as a function of space, to obtain:

$$
\begin{aligned}
-bg(z)(1-z)\rho &= -(b(1-z)+\nu z)\Phi_{\text{in}} + D\frac{\partial^2 \Phi_{\text{in}}}{\partial x^2} \\
0 &= -bg(z)\Phi_{\text{out}} + D\frac{\partial^2 \Phi_{\text{out}}}{\partial x^2}
\end{aligned}
\tag{27}
$$

where $\Phi_{\text{in}}(x,z,L) = bg(z)\rho \int_0^\infty dt\ \phi_{\text{in}}(x,z,t,L)$ and $\Phi_{\text{out}}(x,z,L) = bg(z)\rho \int_0^\infty dt\ \phi_{\text{out}}(x,z,t,L)$, respectively. Note that we have also introduced a new, effective rate of introduction of new species per unit space and time, $bg(z)\rho$, instead of $\nu\rho$, for consistency at small values of L with the term $bg(z)\phi_{\text{out}}$ in Eq. (27). The function $g(z)$ is then fixed by the large scale behavior. In this limit,

$$
bg(z)(1-z)\rho = (b(1-z)+\nu z)\Phi_{\text{in}} \tag{28}
$$

which leads to a large-scale solution

$$
\Psi(z, L\to\infty) = \rho L \frac{\nu}{b-\nu}\log(b/\nu) - \rho L \frac{g(z)(1-z)}{(1-z+\nu z/b)}. \tag{29}
$$

So in order to match the standard neutral result for a well-mixed, non-zero-sum community [1], which is

$$
\begin{aligned}
\Psi(z,L) &= \sum_{k=1}^{\infty} S_{\text{nzs}}(k) z^k \\
&= \sum_{k=1}^{\infty} \frac{\nu\rho L}{b-\nu}\left(\frac{b-\nu}{b}\right)^k z^k \\
&= -\frac{\nu\rho L}{b-\nu}\log\left(1-\frac{b-\nu}{b}z\right)
\end{aligned}
\tag{30}
$$

we need to set

$$
g(z) = \frac{1-z+\nu z/b}{1-z}\frac{\nu}{b-\nu}\log\left(\frac{b-(b-\nu)z}{\nu}\right). \tag{31}
$$

We note also that $g(0) = \nu_{\text{eff}}$, and so this approximation simply reduces to the approximation we used to derive the Species-area Curve above; our solution above for $\phi(z,t,L)$ is equal to $\phi(x, z=0, t, L)$ here, so that our approximation for the Species-area curve satisfies these same equations but with $z=0$ (as it should do).

The result of solving this pair of equations is essentially the same as above. The dependence on z of the parameter does not affect the solution as a function of x, it just changes the parametrization in that solution:

$$
\begin{aligned}
\Phi_{\text{in}}(x,z,L) &= f(z)\rho\left(1 - \frac{\cosh(mx\sqrt{h(z)})}{\sqrt{\frac{1-z}{f(z)}}\sinh(mL\sqrt{h(z)}/2)+\cosh(mL\sqrt{h(z)}/2)}\right) \\
\Phi_{\text{out}}(x,z,L) &= \rho\sqrt{f(z)(1-z)}\frac{e^{-\sqrt{g(z)}m|x-L/2|}\sinh(mL\sqrt{h(z)}/2)}{\sqrt{\frac{1-z}{f(z)}}\sinh(mL\sqrt{h(z)}/2)+\cosh(mL\sqrt{h(z)}/2)}
\end{aligned}
\tag{32}
$$

where again $m = \sqrt{b/D} = 1/\sigma$ in the main text, and for ease of notation we introduce

$$h(z) = \left((1-z) + \frac{\nu}{b}z\right)$$
$$f(z) = \frac{\nu}{b-\nu}\log\left(\frac{b-(b-\nu)z}{\nu}\right) \tag{33}$$

as in the main text, and such that $g(z)$, $f(z)$ and $h(z)$ are related by $g(z) = h(z)f(z)/(1-z)$. We now integrate over space to obtain our approximate prediction for the one-dimensional Species Abundance Distribution.

$$\Psi_{1d}(z,L) = S(L) - \rho f(z)L + \frac{\frac{2\rho}{m\sqrt{h(z)}}(f(z)-(1-z))\sinh(mL\sqrt{h(z)}/2)}{\sqrt{\frac{1-z}{f(z)}}\sinh(mL\sqrt{h(z)}/2) + \cosh(mL\sqrt{h(z)}/2)} \tag{34}$$

The same approach in 2 spatial dimensions for a circular region of radius L and area $A = \pi L^2$ leads to:

$$\Phi_{\text{in}}(r,L) = f(z)\rho\left(1 - \frac{I_0(mr\sqrt{h(z)})}{\sqrt{\frac{1-z}{f(z)}}I_1(mL\sqrt{h(z)})\frac{K_0\left(mL\sqrt{g(z)}\right)}{K_1\left(mL\sqrt{g(z)}\right)} + I_0(mL\sqrt{h(z)})}\right)$$
$$\Phi_{\text{out}}(r,L) = \rho\frac{\sqrt{f(z)(1-z)}\ I_1(mL\sqrt{h(z)})\frac{K_0\left(mr\sqrt{g(z)}\right)}{K_1\left(mL\sqrt{g(z)}\right)}}{\sqrt{\frac{1-z}{f(z)}}I_1(mL\sqrt{h(z)})\frac{K_0\left(mL\sqrt{g(z)}\right)}{K_1\left(mL\sqrt{g(z)}\right)} + I_0(mL\sqrt{h(z)})} \tag{35}$$

and

$$\Psi_{2d}(z,A) = S(A) - \rho f(z)A + \frac{\frac{2(f(z)-(1-z))}{\sqrt{h(z)}}\rho\sqrt{A\pi\sigma^2}\ I_1\left(\sqrt{\frac{h(z)A}{\pi\sigma^2}}\right)}{I_0\left(\sqrt{\frac{Ah(z)}{\pi\sigma^2}}\right) + \sqrt{\frac{1-z}{f(z)}}\frac{K_0\left(\sqrt{\frac{Ag(z)}{\pi\sigma^2}}\right)}{K_1\left(\sqrt{\frac{Ag(z))}{\pi\sigma^2}}\right)}I_1\left(\sqrt{\frac{Ah(z)}{\pi\sigma^2}}\right)}. \tag{36}$$

2.3 Universal behaviour as $\nu \to 0$

Here, we show that the species abundance distributions given by our model exhibit the same universality property found in simulations by Rosindell and Cornell [2] i.e. that the species abundance distributions form a family of curves, parametrised by the single paramemeter $\frac{A\nu}{b\sigma^2}$.

First, we show that the exact solution to the backward equation for ϕ has this scaling property. If

we define

$$
\begin{aligned}
Q &= \frac{b-\nu}{\nu} q \\
T &= \nu t \\
X &= x\sqrt{\frac{\nu}{D}},
\end{aligned}
$$

then eqn. ((7)) becomes

$$
\begin{aligned}
\frac{\partial Q}{\partial T} &= -Q - Q^2 + \left(1 - \frac{\nu}{b-\nu}\right) \frac{\partial^2 Q}{\partial X^2} \\
&\to -Q - Q^2 + \frac{\partial^2 Q}{\partial X^2} \qquad \text{as } \nu \to 0
\end{aligned}
$$

and the initial condition becomes $Q(X, T = 0) = \frac{(b-\nu)(1-z)}{\nu} R(X, L\sqrt{\frac{\nu}{D}})$. Therefore, Q only depends on the parameters through the combinations

$$
\begin{aligned}
Z &= \frac{(b-\nu)(1-z)}{\nu} \\
Y &= \frac{A\nu}{D} = \frac{A\nu}{b\sigma^2}
\end{aligned}
$$

i.e. $Q = \tilde{Q}(X, T, Z, Y)$ for some function $\tilde{Q}$. The generating function for the abundance distribution in 2D is then given by (see eqn. ((23)))

$$
\begin{aligned}
\Psi &= S(A) - \nu\rho \int_{-\infty}^{\infty} \int_{-\infty}^{\infty} d^2x \int_0^{\infty} dt\, \phi(x,t) \\
&= S(A) - \frac{\rho b \sigma^2}{b-\nu} \int_{-\infty}^{\infty} \int_{-\infty}^{\infty} d^2X \int_0^{\infty} dT\, \tilde{Q}(X, T, Z, Y) \\
&= S(A) - \frac{\rho b \sigma^2}{b-\nu} \tilde{\Psi}(Z, Y) \\
&\to S(A) - \rho\sigma^2 \tilde{\Psi}(Z, Y) + O(\nu) \qquad (37)
\end{aligned}
$$

for some function $\tilde{\Psi}$.

While we do not have an expression for the exact solution Ψ, we can verify that our approximate

solution Ψ_{2d} has the same scaling behaviour. Substituting $1-z=\frac{Z\nu}{b-\nu}$, $A=\frac{Yb\sigma^2}{\nu}$ we get

$$
\begin{aligned}
h(z) &= \frac{\nu}{b}(1+Z)+O(\nu^2) \\
f(z) &= \frac{\nu}{b}\log(1+Z)+O(\nu^2) \\
g(z) &= \frac{\nu}{b}(1+Z)\log(1+Z)+O(\nu^2) \\
\frac{f(z)-(1-z)}{\sqrt{h(z)}}\sqrt{A\pi\sigma^2} &= (\log(1+Z)-Z)\sigma^2\sqrt{\pi Y}+O(\nu) \\
\frac{h(z)A}{\pi\sigma^2} &= \frac{Y(1+Z)}{\pi}+O(\nu) \\
\frac{g(z)A}{\pi\sigma^2} &= \frac{Y(1+Z)\log(1+Z)}{\pi}+O(\nu)
\end{aligned}
$$

so eqn. ((36)) becomes

$$
\Psi_{2d}=S(A)-\rho\sigma^2 Y\log(1+Z)+2\rho\sigma^2\frac{(\log(1+Z)-Z)\sqrt{\pi Y}I_1\left(\sqrt{\frac{Y(1+Z)}{\pi}}\right)}{I_0\left(\sqrt{\frac{Y(1+Z)}{\pi}}\right)+\sqrt{\frac{Z}{\log(1+Z)}}\frac{K_0\left(\sqrt{\frac{Y(1+Z)\log(1+Z)}{\pi}}\right)}{K_1\left(\sqrt{\frac{Y(1+Z)\log(1+Z)}{\pi}}\right)}I_1\left(\sqrt{\frac{Y(1+Z)}{\pi}}\right)}+O(\nu)
$$

which is of the same form as eqn. ((37)).

We have thus shown that the generating function of the abundance distribution is a function of two parameter combinations only. We will now show that this is equivalent to the observation that the species abundance distribution is a one-parameter family of curves [2]:

$$
S(k,A)=\nu\tilde{S}\left(\nu k,\frac{A\nu}{b\sigma^2}\right).
$$

(note that the expression in ref. [2] describes the scaling of logarithmic Preston classes of abundance, and hence is missing the prefactor ν). To see how this is related to our scaling expression for $\Psi(z,A)$, we write

$$
\begin{aligned}
\Psi(z,A) &= \sum_{k=1}^{\infty} z^k S(k,A) \\
&\approx \int_1^{\infty} e^{k\log z}\nu\tilde{S}\left(\nu k,\frac{A\nu}{b\sigma^2}\right)dk \\
&= \int_{\nu}^{\infty} e^{m\frac{\log z}{\nu}}\tilde{S}(m,Y)\,dm,
\end{aligned}
$$

where $m=\nu k$. We need to proceed with caution in case $\tilde{S}$ has a non-integrable singlarity in its first argument. Abundance in Preston classes of low order appears from Fig 2 in the paper to

approach a finite limit, so we assume that $\tilde{S} \sim \frac{s(Y)}{\nu k}$ at small (νk). Without loss of generality, we write

$$\begin{aligned}
\Psi(z,A) &= \int_{\nu}^{\infty} \left(\frac{1}{m} s\left(Y\right) e^{-m} + e^{m \frac{\log z}{\nu}} u\left(m,Y\right) - \frac{1}{m} s\left(Y\right) e^{-m} \right) dm \\
&= s\left(Y\right) \mathsf{Ei}(\nu) + f\left(\frac{\log z}{\nu}, Y \right) + O(\nu) \\
&= s\left(Y\right) \mathsf{Ei}(\nu) + f\left(\frac{\log\left(1 - \frac{Z\nu}{b-\nu}\right)}{\nu}, Y \right) + O(\nu) \\
&= s\left(Y\right) \mathsf{Ei}(\nu) + f\left(Z, Y\right) + O(\nu),
\end{aligned}$$

where $\mathsf{Ei}(x) = \int_x^{\infty} \frac{\exp(-y)}{y} dy$ is the exponential integral and f is a (finite) function of two arguments. The component of this expression that depends on Z takes the same scaling form as found above for the backward equation model described above. The term that is independent of Z does not contribute to any particular $S(k,A)$, but does contribute to the total species richness, and indeed we can identify

$$\begin{aligned}
S(A) &= \Psi(1,A) \\
&= s\left(Y\right) \mathsf{Ei}(\nu) + f\left(0, Y\right)
\end{aligned}$$

3 Biological Interpretation of the Approximation Method

By approximating the non-linear term in the defining backward Equation (7) by a heterogeneous linear term, we found the pair of equations (11) to solve for the species area relationship:

$$\begin{aligned}
-b\nu_{\text{eff}}\rho &= -b\Phi_{\text{in}} + D\frac{\partial^2 \Phi_{\text{in}}}{\partial x^2} \\
0 &= -b\nu_{\text{eff}}\Phi_{\text{out}} + D\frac{\partial^2 \Phi_{\text{out}}}{\partial x^2}.
\end{aligned}$$

(For simplicity we work in one spatial dimension but the interpretations are identical in $2d$.) We could equally well interpret these not just as an approximation to Eq. (7), but as a biological model in their own right. If we do so, can we reinterpret these equations and understand biologically why this linear approximation works? Eqs. (11) constitute a system where there is only mortality (driving loss of species from the focal, sample area), dispersal, and input from speciation. This might be expected, since species are only removed from the focal region when there is a mortality event, and only added when there is a speciation event landing in the focal region, or dispersal in from outside. However, in these equations the effective rates of species loss are different for species which originated outside the focal region (rate ν_{eff}), versus those that originated inside the focal region (rate b), and that is what we must explain.

Remembering that in the original derivation above of Eq. (7), the per capita birth rate was $b - \nu$ and mortality rate was b, this interpretation of rate of species loss in the equation for Φ_{in} becomes clear: species are lost from the focal region at the rate at which a single individual dies. I.e. we are approximating that species which originate within $-L/2 < x < L/2$, will only *not* be found at time t in this sample region if it goes extinct, and this rate of loss is approximated by the rate of loss b of a single individual. For species outside the sample region, the rate of loss from mortality is $b\nu_{\text{eff}} = \frac{b\nu}{b-\nu}\log(b/\nu)$. What is this number? In fact, it is equal to $b/\langle n\rangle$, where $\langle n\rangle$ is the expected population size of an extant species (i.e. total number of individuals divided by total number of extant species). On average, a species originating outside the focal region at any point in the past, is lost from the focal region at a effective rate, $b/\langle n\rangle$.

4 Comparison with Field Theory/Forward-in-time Equations

In an earlier paper, one of the authors derived a forward-in-time approach to these same spatial neutral models [3]. In that approach, we also began with the case of a spatially-discrete landscape. Here we will recap the basic features and approximation we made in that paper, and where they break down relative to our current approach. We work here with individuals that diffuse across the landscape, rather than sessile individuals, but as discussed above this does not change the patterns of abundance and occupancy when the speciation rate is small.

We first describe the state of the discrete system using the probability distribution $P(\ldots, n_i \ldots, t)$ that there are n_i individuals at each spatial location, i, at time t, belonging to a focal species. Individuals in this spatially-discrete model die with a per capita mortality rate, d, produce new offspring at a per capita birth rate, b, and may transfer to nearest neighbour cells at a rate $\tilde{D}$. In addition, there is a speciation process modeled as immigration from outside the system at a rate $\tilde{k}$ from 0 to 1 individual:

$$\begin{aligned}\frac{\partial P(\{n_i\},t)}{\partial t} &= d\sum_i (n_i+1)P(\ldots,n_i+1,\ldots,t) - d\sum_i n_i P(\ldots,n_i,\ldots,t)\\ &+ b\sum_i (n_i-1)P(\ldots,n_i-1,\ldots,t) - b\sum_i n_i P(\ldots,n_i,\ldots,t)\\ &+ \tilde{D}\sum_i\sum_{\{e\}}[(n_i+1)P(\ldots,n_i+1,n_e-1,t) - n_i P(\ldots,n_i,n_e,\ldots)]\\ &+ \tilde{k}\sum_i\left(\delta_{n_i 1}\prod_{j\neq i}\delta_{n_j 0}\right) - \tilde{k}\sum_i\left(\prod_j \delta_{n_j 0}\right) \end{aligned} \tag{38}$$

We could also remove this last term, introducing speciation, and thus allow each species to reach permanent extinction. We would then sum the contributions to the present day state from all

species that originated at some point in the past, assuming a uniform speciation rates across time and space. Before taking the limit of continuous space, we rewrite the dynamics of our discrete community in terms of a moment generating function. This generating function is defined by a sum over all spatial configurations of individuals:

$$Z(\ldots, h_i, \ldots, t) = \sum_{\{n_k\}} P(\ldots, n_i, \ldots, t) e^{\sum_j h_j n_j}. \tag{39}$$

Rewriting Eq.(38) in terms of this generating function, we find a new defining equation:

$$\frac{\partial Z}{\partial t} = d \sum_{i=-\infty}^{\infty} \frac{\partial Z}{\partial h_i}\left(e^{-h_i} - 1\right) + b \sum_{i=-\infty}^{\infty} \frac{\partial Z}{\partial h_i}\left(e^{h_i} - 1\right) + \tilde{D} \sum_{i=-\infty}^{\infty} \sum_{\{e\}} \frac{\partial Z}{\partial h_i}\left(e^{h_e - h_i} - 1\right) + \tilde{k} \sum_{i=-\infty}^{\infty} \left(e^{h_i} - 1\right). \tag{40}$$

4.1 Taking a Continuum Limit

We denote the lattice spacing by Δ, and define the continuum limit as follows:

$$\begin{aligned} \sum_i &\to \Delta^{-d} \int d^d x \\ h_i &\to H(x) \\ \frac{\partial}{\partial h_i} &\to \Delta^d \frac{\delta}{\delta H(x)} \\ \tilde{D} &\to \frac{D}{\Delta^2} \\ \tilde{k} &\to k \Delta^d \end{aligned} \tag{41}$$

Finally, to define the continuum limit for the sum over nearest neighbours, we consider a square, d-dimensional lattice:

$$\begin{aligned} \sum_{\{e\}} \left(e^{h_e - h_i} - 1\right) &\to \sum_{k=1}^{d} \left(\exp\left(\Delta \frac{\partial H}{\partial x_k} + \frac{\Delta^2}{2} \frac{\partial^2 H}{\partial x_k^2} + \ldots\right) + \exp\left(-\Delta \frac{\partial H}{\partial x_k} + \frac{\Delta^2}{2} \frac{\partial^2 H}{\partial x_k^2} + \ldots\right) - 2\right) \\ &= \Delta^2 \left(\nabla^2 H(x) + (\nabla H(x))^2\right) + O(\Delta^3) \end{aligned} \tag{42}$$

With these identifications, the multivariate generating function Eq. (39) becomes a functional of the source $H(x)$:

$$Z[H(x), t] = \left\langle e^{\int dx H(x) n(x)} \right\rangle \tag{43}$$

where $\langle n(x) \rangle$, $\langle n(x_1) n(x_2) \rangle$ etc are expectation values of the number densities and correlations of individuals as a function of spatial location (express this better). This generating functional

satisfies the continuum limit of Eq. (40), the following functional differential equation:

$$\frac{\partial Z}{\partial t} = d\int d^dx \frac{\delta Z}{\delta H(x)}\left(e^{-H(x)}-1\right) + b\int d^dx \frac{\delta Z}{\delta H(x)}\left(e^{H(x)}-1\right)$$
$$+ D\int d^dx \frac{\delta Z}{\delta H(x)}\left(\nabla^2 H(x) + (\nabla H(x))^2\right) + kZ\int d^dx\left(e^{H(x)}-1\right)$$
$$= \int d^dx\left(e^{H(x)}-1\right)\left(-de^{-H(x)}\frac{\delta Z}{\delta H(x)} + b\frac{\delta Z}{\delta H(x)} + D\nabla^2\left(e^{-H(x)}\frac{\delta Z}{\delta H(x)}\right) + k\right). \quad (44)$$

where we have performed an integration by parts and assumed that the source $H(x)$ vanishes at infinity. Finally, we make a change of variables for the source,

$$J(x) = e^{H(x)} - 1 \quad (45)$$

so that $\mathcal{Z}[J(x), t] = Z[\log(J(x)+1), t]$ satisfies

$$\frac{\partial \mathcal{Z}}{\partial t} = \int d^dx\ J(x)\left((b-d)\frac{\delta \mathcal{Z}}{\delta J(x)} + bJ(x)\frac{\delta \mathcal{Z}}{\delta J(x)} + D\nabla^2\frac{\delta \mathcal{Z}}{\delta J(x)} + k\right). \quad (46)$$

4.2 Equal Time Correlation Functions

The n-point spatial correlation functions for this model, taken at equal times, t, satisfy a set of partial differential equations. These equations are obtained by expanding $\mathcal{Z}[J(x), t]$ as a functional Taylor series:

$$\mathcal{Z}[J(x), t] = \int d^dx\ c_1(x,t)J(x) + \frac{1}{2}\int d^dx_1 d^dx_2\ c_2(x_1, x_2, t)J(x_1)J(x_2) + \ldots \quad (47)$$

The coefficients of this Taylor series are obtained by taking functional derivatives of Eq.(46) with respect to J, and then setting $J = 0$. For the first two orders we have:

$$\frac{\partial c_1}{\partial t} = (b-d)c_1 + D\nabla^2 c_1 + k$$
$$\frac{\partial c_2}{\partial t} = 2bc_1\delta(x_1 - x_2) + D\left(\nabla_1^2 + \nabla_2^2 + 2(b-d)\right)c_2(x_1, x_2, t) \quad (48)$$

Each successive order relies only on solutions for correlation functions of lower order, and so the system of linear partial differential equations can be solved exactly, given a set of initial data.

4.3 Species Area Relationship

We now consider the time-independent probability $P(N, L)$ that at late times there are N individuals in a given sample region extending from $-L$ to $+L$. The generating function of this probability

is:

$$\psi(j,L) = \sum_{N=0}^{\infty} P(N,L)(1+j)^N = \left\langle e^{\log(1+j)\int_L dx\ n(x)} \right\rangle. \tag{49}$$

To underline the interpretation: $P(N,L)$ is the (assumed time-independent solution for the) probability distribution that we will find N individuals in the region between $-L$ and $+L$ at late times. The second equality arises because this generating function can be obtained by setting $J(x) = j\text{Rect}(x,L)$ in the late-time, time-independent solution for $Z[J,t]$, where Rect(x,L) is the rectangular function in 1d. I.e. we will set $J(x)$ zero outside the sample region and equal to j inside. The expected number of species in the sampling region defined by L is proportional to $1-P(0,L)$, and so this is the quantity we are aiming to solve for. If we can solve for this generating function then we have $P(0,L) = \psi(-1,L)$. This is also known as the empty interval function, e.g. [4]. To find $\psi(-1,L)$, we next define the modified moments which have insertions of $e^{\log(1+j)\int_L n(x)}$ compared with the usual moments:

$$\begin{aligned} f_1(x,j,L) &= \left.\frac{\delta\mathcal{Z}[J]}{\delta J(x)}\right|_{J=j\text{Rect}(x,L)} \\ &= \frac{1}{1+j\text{Rect}(x,L)}\left\langle n(x)e^{\log(1+j)\int_L n(x)}\right\rangle \end{aligned} \tag{50}$$

$$\begin{aligned} f_2(x,y,j,L) &= \left.\frac{\delta^2\mathcal{Z}[J]}{\delta J(x)\delta J(y)}\right|_{J=j\text{Rect}(x,L)} \\ &= ... \end{aligned} \tag{51}$$

We note that the reason for using these functions is that:

$$\frac{\partial\psi}{\partial j} = \frac{1}{1+j}\left\langle \int_L dx n(x) e^{\log(1+j)\int_L n(x)}\right\rangle = \int_L dx f_1(x,j,L). \tag{52}$$

and hence if we can solve for $f_1(x,k,L)$ we will have the empty interval function, $P(0,L)$.

We can obtain differential equations for these modified moments by taking successive functional derivatives of Eq.(46):

$$\begin{aligned} \frac{\partial}{\partial t}\frac{\delta\mathcal{Z}[J,t]}{\delta J(x)} &= D\nabla^2\frac{\delta\mathcal{Z}}{\delta J(x)} + (bJ(x)+b-d)\frac{\delta\mathcal{Z}}{\delta J(x)} + k \\ &\quad + \int dy\ J(y)\frac{\delta}{\delta J(x)}\left[D\nabla^2\frac{\delta\mathcal{Z}}{\delta J(y)} + (bJ(y)+b-d)\frac{\delta\mathcal{Z}}{\delta J(y)} + k\right] \\ \frac{\partial}{\partial t}\frac{\delta^2\mathcal{Z}[J,t]}{\delta J(x)\delta J(y)} &= ... \end{aligned} \tag{53}$$

etc. So setting $J(x) = j\text{Rect}(x,L)$ and time derivatives equal to zero in these equations we have a kind of moment hierarchy:

$$\begin{aligned} 0 &= D\nabla_x^2 f_1(x,j,L) + (bj\text{Rect}(x,L)+b-d)f_1(x,j,L) + k \\ &\quad + j\int_L dy\ \left[D\nabla_y^2 f_2(x,y,j,L) + (bj+b-d)f_2(x,y) + b\delta(x-y)f_1(y,j,L)\right] \end{aligned} \tag{54}$$

$$0 = D\nabla_x^2 f_2(x,y,j,L) + ... \tag{55}$$

So far there is no approximation. In our earlier paper we truncated and solved the first equation in this hierarchy:

$$0 = D\nabla^2 f_1(x, j, L) + (bj\text{Rect}(x, L) + b - d)f_1(x, j, L) + k \tag{56}$$

While giving qualitatively accurate description of the shape of the SAR, this earlier approximation becomes quantitatively inaccurate as a speciation rate becomes small. It also fails to give a good description of the Species Abundance Distribution.

5 Numerical Inversion of the SAD Generating Function

We implemented a method described in [5] for numerical contour integration using Cauchy's theorem. Our annotated and documented code will be freely available on the O'Dwyer lab GitHub repository.

6 Uncertainty in Estimating Speciation Rates

In our analysis of empirical data showin in Fig.3 of the main text, we draw from earlier work fitting speciation rate ν and dispersal length-scale σ using [6] using multiple 1ha plots distributed across Panama. One other important conclusion from this work was that the best fit parameter for ν was highly variable across different locations (e.g. in comparison with Panama, two South American locations were fitted with speciation rates that were orders of magnitude smaller). Here, we repeat the analysis of Figure 3, but using one of these lower fitted speciation rates (and the corresponding value of σ, alongside the local density of individuals in Ecuador). Clearly, this cannot be a complete analysis, as we are no longer providing the best fit to the large-scale Panamanian data. But it is interesting to ask whether these much lower speciation rates can significantly change either the local prediction for overall species richness, or whether the shape of the local SAD becomes more realistic. We show this analysis in Fig S1. The result is that we do find a larger expected species richness locally, but still not close to the observed number of species at BCI. At the same time, the shape of the species abundance distribution is even more skewed away from rare species.

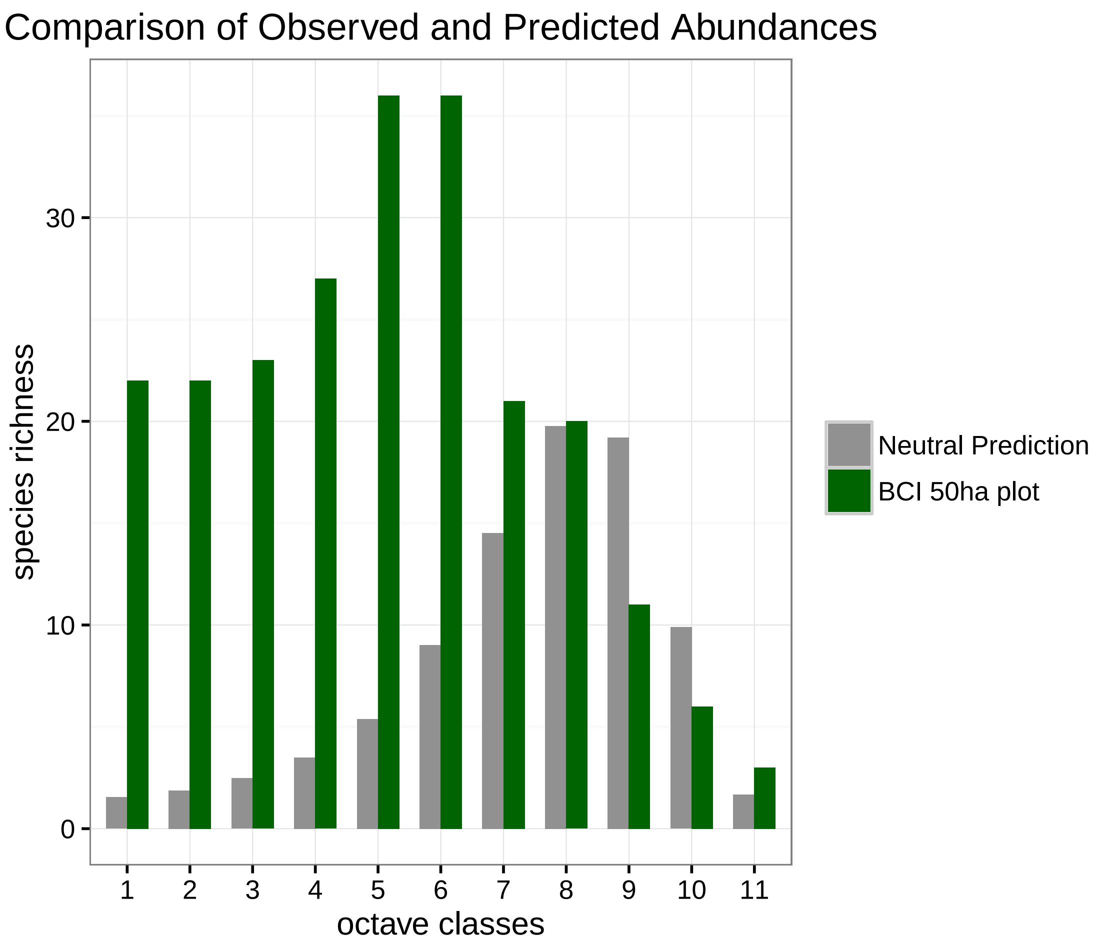

Figure S1 **Neutral predictions at BCI, using Yasuni fitted parameters**. Neutral predictions are generated by fitting our spatial neutral model using large-scale data reported and analyzed in [6], but now using the lower reported speciation rate fitted using data surrounding the Yasuni CTFS plot in Ecuador ($\nu = 1.7.10^{-11}$, rather than $\nu = 5.10^{8}$), alongside the other parameters σ and density ρ fitted at Yasuni (which were of the same order of magnitude as those in Panama). The results show that these large-scale fits still produce a local-scale prediction for species abundances that both underestimates local species richness, compared with observed data, and still skews abundances from rare to more abundant species.

References

[1] I. Volkov, J. R. Banavar, S. P. Hubbell, and A. Maritan. Neutral theory and relative species abundance in ecology. *Nature*, 424:1035–1037, 2003.

[2] James Rosindell and Stephen J Cornell. Universal scaling of species-abundance distributions across multiple scales. *Oikos*, 122(7):1101–1111, 2013.

[3] JP O'Dwyer and JL Green. Field theory for biogeography: a spatially-explicit model for predicting patterns of biodiversity. *Ecology Letters*, 13:87–95, 2010.

[4] C. Doering and D. Ben-Avraham. Diffusion-Limited Coagulation in the Presence of Particle Input: Exact Results in One Dimension. *Phys Rev Lett*, 62:2563–2566, 1989.

[5] Folkmar Bornemann. Accuracy and stability of computing high-order derivatives of analytic functions by cauchy integrals. *Foundations of Computational Mathematics*, 11(1):1–63, 2011.

[6] R. Condit, N. Pitman, E.G. Leigh, J. Chave, J. Terborgh, R.B. Foster, P. Nunez, S. Aguilar, S. Valencia, G. Villa, H.C. Muller-Landau, E. Losos, and S.P. Hubbell. Beta-diversity in tropical forest trees. *Science*, 295(5555):666–669, 2002.
